# An analysis of reported motivational orientation in students undertaking doctoral studies in the biomedical sciences

**DOI:** 10.1186/1472-6920-14-38

**Published:** 2014-02-27

**Authors:** Matthew W Kemp, Timothy J Molloy, Marina Pajic, Elaine Chapman

**Affiliations:** 1 School of Women’s and Infants’ Health, M550, The University of Western Australia, Perth WA6009, Australia; 2 Graduate School of Education, M428, The University of Western Australia, 35 Stirling Highway, Crawley, Perth WA 6009, Australia; 3 The Kinghorn Cancer Centre and Cancer Research Program, Garvan Institute of Medical Research, Sydney, Australia

**Keywords:** Ph.D., Biomedical, Self-determination theory, Motivation, Doctorate, Control

## Abstract

**Background:**

As the source of a sizeable percentage of research output and the future arbiters of science policy, practice and direction, doctoral (Ph.D.) students represent a key demographic in the biomedical research community. Despite this, doctoral learning in the biomedical sciences has, to date, received little research attention.

**Methods:**

In the present study we aimed to qualitatively describe the motivational orientations present in semi-structured interview transcripts from a cohort of seventeen biomedical Ph.D. students drawn from two research intensive Australian Group of Eight universities.

**Results:**

Applying elements of self-determination theory, external and introjected control loci (both strongly associated with alienation, disengagement and poor learning outcomes) were identified as common motivational determinants in this cohort.

**Conclusions:**

The importance of these findings to doctoral learning is discussed in light of previous research undertaken in higher education settings in the United States and the European Union. With motivation accepted as a malleable, context-sensitive factor, these data provide for both a better understanding of doctoral learning and highlight a potential avenue for future research aimed at improving outcomes and promoting meaningful learning processes in the biomedical doctorate.

## Background

The discipline of doctoral education has received increasing attention over the past two decades. This increase in research activity is due, in part, to large increases in student enrolments in doctoral programs and demands for greater regulatory oversight and accountability from government agencies that fund universities. However, perhaps the greatest stimulus for research into the practice of doctoral education is the sizable percentage of doctoral students who fail to complete their studies.

A number of (predominantly US-based) studies exist to demonstrate that between 30 and 50 % of doctoral candidates fail to complete their degrees [1,2]; the highest rates of attrition reported are in the humanities and social sciences with the lower rates of attrition in the biological sciences and in programs undertaken in professionally-orientated (medicine, business, law) faculties [3]. In Australia, a similar pattern is apparent [4]. In 1999, Martin and colleagues estimated that of the cohort of Ph.D. students who enrolled in 1992, only 61.6 % would complete their studies by 2003 [5]. These data are supported by a more recent study by Jiranek, which suggests an attrition rate of 33 % in dissertation research students enrolled in the Faculty of Sciences at the University of Adelaide [6].

Despite these contemporary developments, relatively little specific focus has been applied to the scholarship of doctoral learning in the biomedical sciences [7-10]. As discussed by Golde and others, significant differences are found in the socio-cultural and operational construction of individual academic disciplines [2]. In an attempt to contribute to the body of data describing the form and function of doctoral learning in the biomedical sciences, we undertook a series of semi-structured interviews with students enrolled in two Australian Group of Eight (Go8) Universities. As discussed below, a thematic analysis of student responses found that learning relationships, learning objectives (both dealt with in separate submissions) and motivation were perceived as key elements of the learning environment. In the present submission, we used elements of self-determination theory to analyse the identified theme of reported motivational orientations.

Previous research has highlighted issues relating to student socialisation [11,12], success at high school [13], supervision and support [1,14,15], race [16,17] and funding [18] as factors influencing doctoral success. Of late, the issue of student motivation has also begun to receive increasing interest as an important component of doctoral learning [3,19,20]. A role for motivation in determining learning success is perhaps unsurprising; the phenomenographic reduction of learning is represented by three elements: the indirect object (the motivation or aim for engaging in the process of learning); the direct object (what is being learnt); and the act (the functional mechanics of the learning process itself) [21-23]. In addition to comprising one element of learning as a phenomenon, a substantial body of data now exists to demonstrate that a student’s motivational orientation also greatly impacts on the remaining two phenomenographic elements of learning, namely what is learnt and how the student engages in the learning process itself [19,20,24]. Despite this, little empirical data exists to describe the motivational orientations of doctoral students working in the biomedical sciences.

### Operational definition of motivation

Motivation is a key element in explaining human behaviour or, as described by Forbes (p.85) “why we do the things we do the way that we do them” [25]. As noted by Ryan and Deci (p.54), motivation can be assessed both quantitatively (as a measure of one’s enthusiasm for a particular task) and qualitatively (as an assessment of the beliefs that give rise to a task being undertaken) [26]. In the present study, we restricted our assessment to a qualitative analysis of students’ motivational orientation. Two elements of self-determination theory, cognitive evaluation theory and organismic integration theory were employed as a theoretical framework to evaluate students’ intrinsic or extrinsic motivational regulation [26-29].

### Intrinsic motivation, extrinsic motivation and self-determination theory

Intrinsic motivation (IM) and extrinsic motivation (EM) are core elements of cognitive evaluation theory and organismic integration theory; two components of self-determination theory (for a comprehensive review of the development of self-determination theory, including cognitive evaluation and organismic integration theories see Vansteenkiste, *et al*. and references therein [29]. IM is proposed as an inherent human trait; intrinsically motivated activities are defined as (p. 233) “those that individuals find interesting and would do in the absence of operationally separable consequences” [30]. In contrast, EM (the form of regulation recognised in Skinner’s operant theory [31]) is characterised by behaviour controlled by external contingencies under which a task is performed to obtain reward or benefit external to the task itself [26,30].

In cognitive evaluation theory, IM is viewed as a (p.56) “significant feature of human nature that affects performance, persistence and well-being across life’s epochs” that is predicated upon the need for relatedness, autonomy and competence [26,30,32]. A significant body of experimental data exists to demonstrate that in learning environments, IM is supported by positive feedback, and perceptions of competence, relatedness and autonomy. A high IM is accompanied by improvements in motivated persistence, task performance and well-being [26,29,30].

Interestingly, the introduction of extrinsic contingencies (material and/or symbolic rewards, control, surveillance and competition) is demonstrated to associate with undermining of IM resulting in impaired performance, alienation, and task disengagement [32].

For some time, IM and EM were viewed as separable and opposing constructs [29]. An important contribution of organismic integration theory (the second self-determination sub-theory) derives from a series of empirical observations demonstrating that EM can be perceived by an individual as either completely external or autonomous (i.e. internalised) to a varying degree [32,33]. Internalisation of EM occurs via a process wherein the individual develops a sense of personal affiliation with, and autonomy over, a particular contingency, personally endorsing both its significance and value [29,30,33]. Interestingly, increasing internalisation has been demonstrated to promote improved task persistence, engagement in addition to perceptions of competence, relatedness and autonomy [29,32,33]. As noted by Fazey (p. 347) “a student who is studying only in order to achieve a better job, and who is not interested in the degree *per se*, would score high on external regulation” [34].

### Motivation and learning in higher education

Both qualitative and quantitative elements of motivation have been identified as important factors in shaping student learning approaches and outcomes in undergraduate learning [20,22,24,35-37]. Building on the phenomenographic reduction of learning [21,23,38], analytical instruments including Biggs’ Study Process Questionnaire (SPQ) [35] and the Approaches and Study Skills Inventory for Students (ASSIST) place importance on motivation in investigating student learning approaches. In Biggs’ SPQ model, EM is reflected in students adopting a surface orientation to their learning. Similarly, factors characterised by EM (lack of purpose, lack of understanding, fear of failure) are highlighted in students scoring highly for a surface apathetic approach to learning in Entwistle, Tait and McCune’s ASSIST instrument [39].

Highlighting the individual, context-sensitive nature of motivation in learning, Fazey and Fazey used the Academic Motivation Scale (see Vallerand *et al.* 1992) to investigate motivations for studying in a cohort of 394 Welsh students beginning their first year of university. In this cohort, mature-age students scored higher for IM than younger students, and younger students scored higher for identified and external regulation [34,40]. Heikkila and Lonka investigated learning approaches, learning regulation and achievement strategies in a cohort of 366 students and demonstrated that external regulation of learning/EM clustered with a surface approach to learning, regulation problems and task irrelevant behaviour [41]. Similar findings were reported by Kember, who identified poor academic achievement and a surface approach to learning in a student lacking intrinsic interest in his Hong Kong-based studies [24]. More recently, Kyndt *et al.* employed self-determination theory to investigate the direct and indirect effect of motivation of student approaches to learning and concluded that autonomous motivation (scoring high for IM) correlates positively with meaningful (deep or extended abstract) approaches to learning and negatively with surface approaches [20]. Interestingly, a subsequent study by the same group demonstrated that motivation was more strongly correlated with the adoption of a deep approach to learning than a student’s working memory capacity (the ability to handle and retain information whilst multi-tasking or dealing with distraction), further underscoring the importance of motivation to student learning approaches [36].

A smaller number of studies have also looked specifically at motivation in various aspects of doctoral learning, and demonstrated a similarly important role in promoting meaningful, deep approaches to learning [1,3,19,42]. Brailsford interviewed a cohort of 11 history Ph.D. graduates to ascertain their motivations for commencing doctoral study and identified internalised (intrinsic interest and personal development) as well as external (improvement of career prospects) elements, concluding (p. 15) that universities would do well to offer “workshops for would-be candidates before enrolment so that initial motives for doctoral study can be explored and reflected upon before a candidate embarks” [19]. Similarly, Castro and colleagues interviewed 7 female doctoral students in the field of counselling, with the aim of obtaining an understanding of the experiences that led them to undertake a Ph.D. External (obtain a better job, avoiding poverty) or partially internalised (demonstrating aptitude or ability to others) motivations were reported by 6 of 7 respondents in the cohort. An analysis of learning approaches and outcomes adopted by this cohort was not provided, however it is interesting to note that the authors reported that the study participants used their previous adversity (poverty, etc.), exhibiting marked external or introjected control loci, as a positive motivational tool and reported a strong sense of autonomy and agency in their studies [43].

Interestingly, a survey instrument-based study of 125 doctoral students by Mason reported a positive relationship between autonomy and motivation to continue (persistence) in their studies [3]. Motivation is also established as key to the success of students completing their doctorates and transitioning to the role of an independent researcher. Lovitts notes that (p.313) “their motivation during the independent stage…is an important determinant of whether they will actually finish their research and their dissertations, and the nature and quality of the contribution they make” and that (p. 315) “students who do not have a strong interest in, or ideas about, their project not only have a harder time with the transition but also produce lesser quality dissertations” [42].

## Methods

### Research questions

This study forms a subset of a wider analysis of student perceptions of motivation (the present analysis), peer relationships and perceived learning objectives (both dealt with separately) in the doctoral learning environment. These inductive themes were identified in an initial analysis of interview transcripts. Using elements of self-determination theory, this study aimed to: **i)** describe the motivational orientations (attitudes, beliefs and goals underpinning students’ attempts to complete a Ph.D.) reported by doctoral students working in the biomedical sciences at two Group of Eight Universities in Australia; and **ii)** determine the extent to which students attitudes, beliefs and goals in undertaking doctoral research in the biomedical sciences represented intrinsic and/or extrinsic motivational regulation.

### Methodology

This study is based on of a series of semi-structured interviews to investigate students’ attitudes, beliefs and goals towards their doctoral studies in the biomedical sciences [9,10]. Semi-structured interviews are used extensively in education and social sciences contexts, [44,45]; the use of pre-set questions in a semi-structured fashion by an interviewer allows for the capture of precise data whilst retaining scope for participants to answer questions or diverge to topics felt to be of individual relevance or importance.

We aimed to enroll 20 Ph.D. students studying in the biomedical sciences at two research-intensive Group of Eight (Go8) Universities in Australia (henceforth referred to as Institution A and Institution B). The Go8 represents the eight highest-ranked teaching and research universities in Australia, receiving the bulk of research funding provided by the Australian Federal Government’s National Health and Medical Research Council [46,47]. We classified biomedical sciences as encompassing the natural, life and health sciences; the uniting factor was that research undertaken in these areas was done with the overarching aim of improving human health and wellbeing by allowing for the development of treatments, preventions or cures of human diseases.

Following review and approval from the Human Research Ethics Committees from both institutions, participants were recruited to the study via solicitations delivered over department email lists and by personal referral. 17 students (Institution A, *n*=10; Institution B, *n*=7) were recruited. All interviews were recorded. Informed consent was obtained, in writing, from each participant before interviews commenced. Prior to interview, participants were informed that the investigators wished to understand more about what they found to be important in their learning as doctoral students. All participants were provided with a debriefing as to the purpose of the study and the methodology employed at the conclusion of their interview. All participants were advised of their right to withdraw from the study without prejudice. All responses were transcribed verbatim by the interviewer within one week of the interview taking place. Grammatical errors were not corrected and participant intonation (pauses, emphasis) were noted in the transcripts. Transcript fidelity was assessed by comparing text with 3, randomly selected 10 second excerpts of the interview recording and all transcripts were de-identified prior to analysis taking place.

65% of study participants were female and the majority (88%) of participants were domestic enrolments. 3 students were in their first year of study, 5 in their second year of study, 6 in their third year of study and 2 students had been enrolled for more than four years. One study participant did not report their candidacy status. The average interview length was 35.5 minutes. Students reported working in a range of biomedical fields including: physiology, cancer biology, musclo-skeletal disorders, reproductive biology, pharmacology, and cell signalling.

Transcripts were initially analysed using a notation/memoing approach described by Miles and Huberman, to identify minor and repetitive themes from responses in single interview transcripts and across the interview set as a whole [45]. Having identified motivation as a major theme across interview transcripts we then employed elements of self-determination theory to re-analyse these data. This process led to the identification of three distinct motivational orientations presented below.

In the present study, the interviewer (34 years old, male, biomedical scientist with experience in supervision and an interest in doctoral education) adopted a non-evaluative, interested listening approach to the conduct of interviews [48]. Key questions in the present analysis of motivational orientation were: **i) ** What do you see as the purpose or purposes of a doctoral program in the biomedical sciences; **ii) ** Please discuss your approach to learning; **iii) ** Why did you decide to do a Ph.D.; **iv) ** How and why did you choose your topic; **v) ** How do you stay motivated; **vi) ** Do you think your Ph.D. is of value; **vii) ** What are your aims for when you have finished studying; and **viii) ** What are some of the positive outcomes that might stem from your Ph.D.

## Results

**Figure 1:**
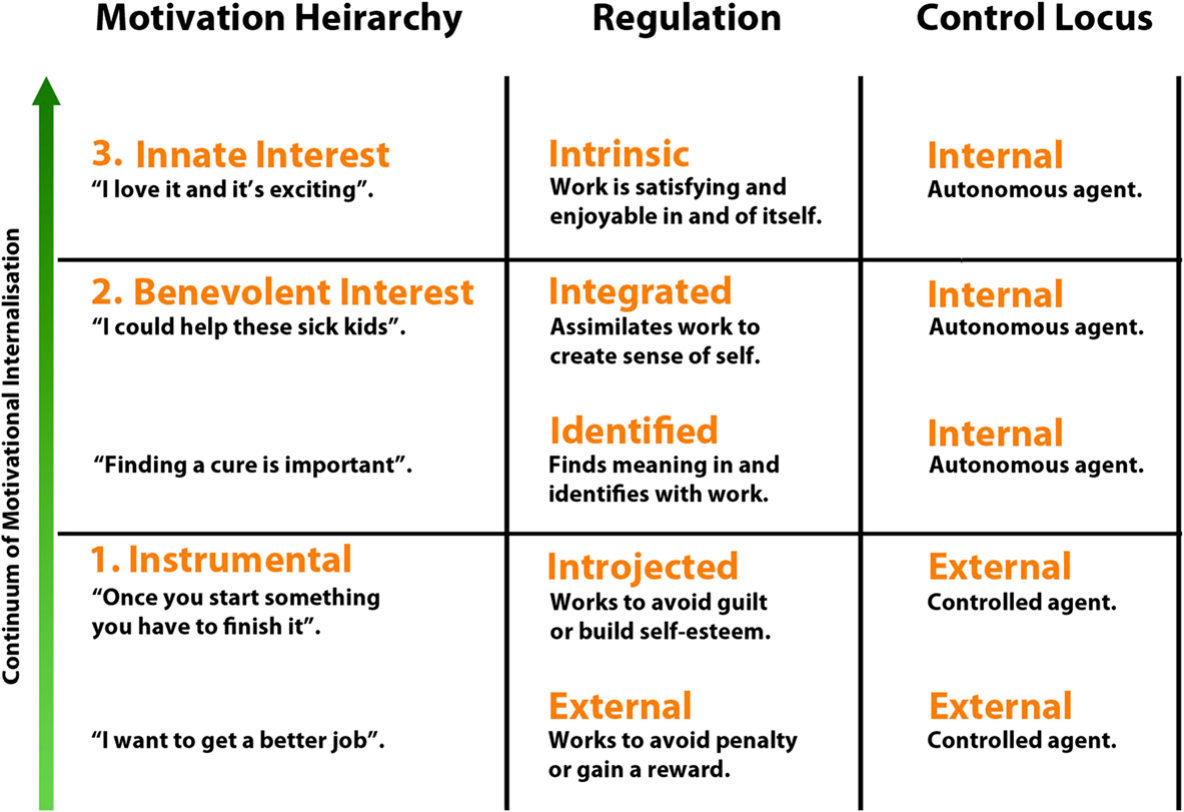
**Continuum of EM internalisation across motivational orientations identified in doctoral students at two institutions.** Structure of figure adapted from Self Determination Theory propounded by Ryan and Deci [26,28].

An analysis of student motivations inductively identified in interview transcripts, using self-determination theory, demonstrated three distinct motivational orientations common to students studying at Institution A and Institution B: **i) ***Instrumental:* where students’ work was undertaken to obtain reward or avoid guilt, demonstrating EM or introjected motivation and an external control locus; **ii) ***Benevolent Interest*: where students’ work was undertaken due to personal identification with the research topic at hand, demonstrating a high degree of internalisation and an internal locus of control; and **iii) ***Innate Interest*: where students’ work was undertaken due to an innate interest in their research and was driven by IM and an internal locus of control. Figure 1 represents a hierarchical summary of the three identified motivational clusters. ‘[…]’ represents transcript excerpts that have been redacted to prevent identification of study participants.

### Instrumental

An instrumental approach was the most commonly reported motivational orientation in our cohort. 16 out of 17 students reported undertaking their doctoral studies for reasons consistent with EM and an external or introjected locus of control. Obtaining an extrinsic, separable reward in the form of a better job or lifestyle was the most commonly reported motivation for undertaking doctoral research in this classification and is suggestive of EM with an external locus of control.

*STUDENT B*: Lifestyle. So the main reason was really that I wanted to secure an area or niche or career path for myself away from the traditional pharmacy career paths. Career. I think you go to university. To obtain a qualification. To get a job. And the better the qualification the better the job, the better the pay, the better the lifestyle.

*STUDENT E*: At this stage I want to stay in science, so yes, you can’t be a postdoc without doing a Ph.D., so it is one of the necessary steps in this career path.

*STUDENT I*: Well there are the skills that I have developed and the opportunities that it affords me. I think those, the career opportunities, the skills interpretation and analysis.

*STUDENT K*: Well… I was working here already. It just seemed like the next logical step I guess. Because the project did interest me and switching from being an RA to being a PhD wasn’t that much of a big change particularly if you were working in the same group.

*STUDENT O*: So as a clinician it is looked on favourably having publications behind you, it helps you get jobs, it helps you if you want to become a P.I. on a clinical trial, patients will often find you through the things you have done in research. It helps with funding if you want to get funding for anything else, it helps you teach if you have a research background and if I decide I want to do research as part of my work.

*STUDENT P*: And I guess the lifestyle as well. I had a toddler and that stage and another baby on the way and it also fitted in well with that, more time at home and not on call.

*STUDENT Q*: Um, for myself, hopefully a good job and some good papers to help me with my science career and papers are really important for funding. So that’s about it. An overseas job as well.

Undertaking research to build self-esteem, obtain external validation from peers/society or continuing with research to avoid failure is consistent with introjected EM and was the second most commonly reported motivation identified in our Instrumental classification. Student A (below) reports being motivated to finish her studies due to the influence of a parent Several students indicated that being awarded a qualification (Student D) or the title of doctor (students H and J) was an important motivation for undertaking a Ph.D.

*STUDENT A*: I think, I don’t know, I think, because I was a kid, my mum said, once you start something you have to finish it. So this sentence make me, well I start my Ph.D. If I can go back and think whether I want to do Ph.D. or not, maybe I won’t do Ph.D. But because I’ve started it, I will finish it.

*STUDENT D*: Even if I don’t become a career academic it is a qualification and probably the highest qualification that I am ever likely to get so it is very valuable for me personally.

*STUDENT H*: Um, I guess it is being a doctor. That, I think you’d find that if you didn’t have that there would be a lot less Ph.D. students. It does put you that step above people around you who haven’t done a Ph.D. Being a doctor. And I don’t think many people go into it saying I am going to learn as many skills as I can and I didn’t think that either. I think it was Just to get ahead.

Student H was then asked to expand on his or her response:

*Interviewer*: So you saw the purpose of a Ph.D. as a mechanism for advancing career and a bump up in social status?

*STUDENT H*: Yep, which is terrible when I think about it.

*STUDENT J*: Even if you don’t go along the academic path I think it is a good process and it is a title as well.

### Benevolent interest

Our second classification of motivational orientation, Benevolent Interest, was characterised by responses demonstrating that students had comprehensively internalised an EM. 8 out of 17 students reported a motivational orientation classified as Benevolent Interest. In this classification, students had found personal, validated meaning in the work that they were undertaking, and incorporated it into their sense of self such that they were working as an autonomously controlled agent. Responses were characterised by a desire to improve the lot of others, based upon a personal commitment or value assessment of the work being undertaken as part of their doctoral studies.

*STUDENT F*: My supervisor is an [*occupation*] so you get to see the kids [*location*] and you can really see that you are doing this for a good purpose and it really motivates you and it is really exciting and I love it. To really make a difference, I think that is really exciting and to expand knowledge in the field I think that is good.

*STUDENT G*: I want to go to university because I want to educate the younger generation as a lecturer, I want to inspire them. I think the education in my country is still maybe 20–30 years backdated from what we have here. Students are mostly spoon fed, I used to sit in a lecture theatre from 8–6 and there was no independent learning at all. I want to change that. Maybe we can have more independent students and people who can be marketed to an international level.

*STUDENT I*: And my research there is the potential that my research could influence clinical practice and I think there is the likelihood that it will influence clinical practice in [*discipline*]. And then it may spread from there.

*STUDENT Q*: Hopefully a cure for cancer – maybe a new treatment or a new biomarker. This protein is really interesting that I am working on and has a lot of potential.

### Innate interest

11 out of 17 student responses contained elements that were classified as demonstrating innate interest in their research. In this classification, motivational orientation was characterised by an intrinsic interest in, and excitement about, the work being undertaken, with involvement in the research project constituting an inseparable reward. The Innate Interest motivational orientation is consistent with students acting as autonomous agents in their learning. A key distinguishing factor between the Innate and Benevolent Interest classifications is the concept of research translation. In the Benevolent Interest category, motivation derives from a desire to see research translated into something that provides a tangible benefit to an individual or society. This feature was absent from the Innate Interest classification, where the process of research discovery was regarded as interesting in and of itself.

*STUDENT D*: One, I think because I liked the area, I liked doing research, I liked discovering research and doing things that no one else has been able to do. I’m still not absolutely certain that I want to be a career academic, but I thought this was a good opportunity since, to do it.

*STUDENT E*: It’s because it was something that I was interested in. When I finished my undergrad. degree I wanted to do Hons. because I was interested in seeing about scientific research and it was a project that I was interested in.

*STUDENT N*: Um, I have always wanted to do research, it is always something that I have been interested in.

*STUDENT P*: I always had an interest in science, in sort of understanding the mechanisms underlying clinical diseases and really the opportunity came through meeting my supervisor in my last year of training and thinking that if I don’t do it now I never will. I found it rewarding myself.

*STUDENT Q*: ‘Cos I really like science, that was mainly it. And I couldn’t see myself doing anything else and I really like being in the lab.

### Distribution of motivational orientations in the study population

**Figure 2:**
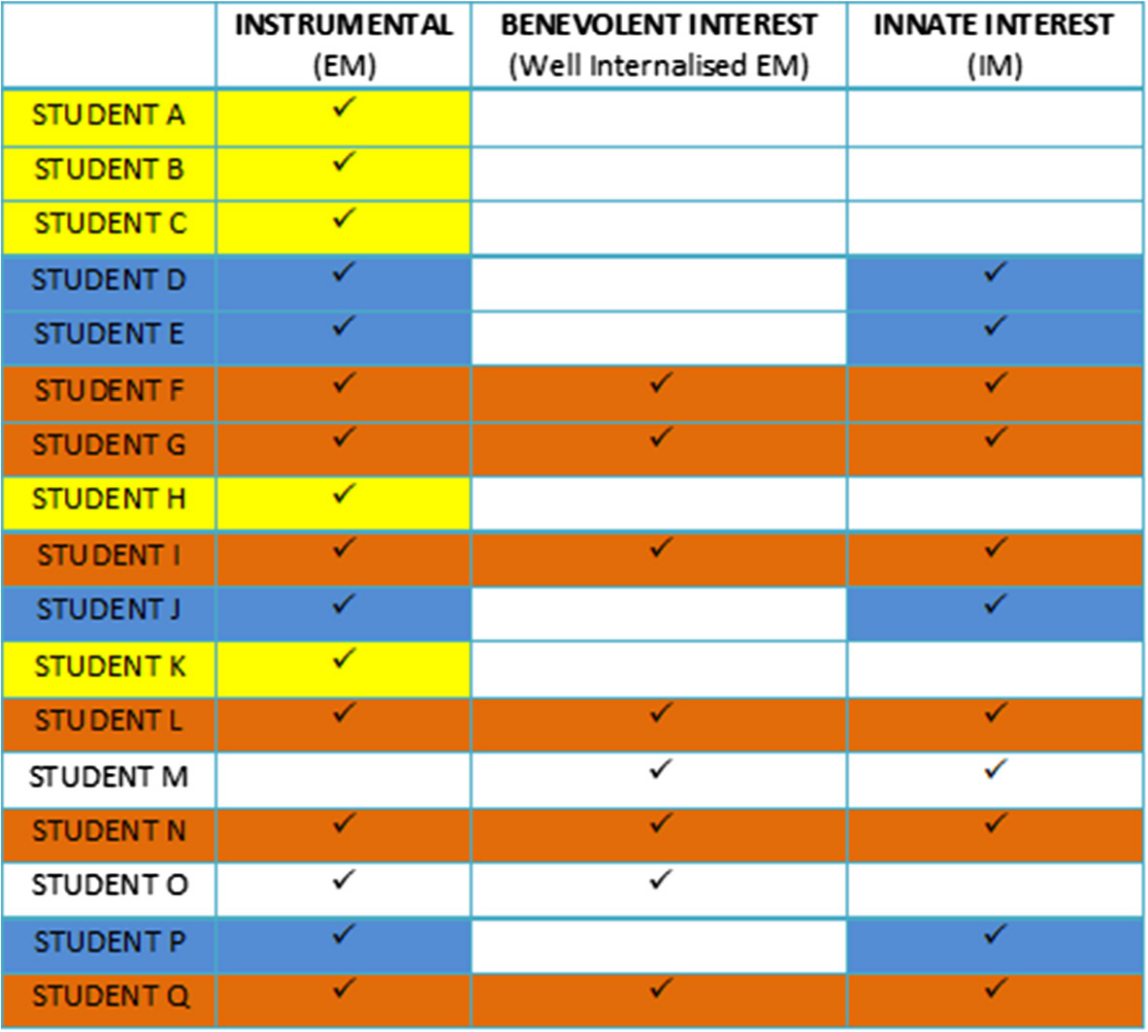
Stratification of motivational orientations across individual students.

Stratifying motivational orientations by individual students allowed us to identify three groups of reported motivational orientation in our study cohort (Figure 2). The first group (*n*=5, highlighted in yellow) was characterised by students who reported only an instrumental motivational orientation (A,B,C,H,K) to their studies, characterised by an external locus of motivational control. In the second group, students (D,E,J,P) reported both self-interest and innate interest (*n*=4, highlighted in blue) as motivational orientations, characterised by a dual externally regulated motivation and IM. Students (*n*=6, highlighted in orange) in the third grouping (F,G,I,L,N,Q) identified self-interest, benevolent interest and innate interest as motivational orientations for undertaking a Ph.D. Responses from two students (M, reporting only benevolent interest and innate interest and O, reporting only self-interest and benevolent interest) did not map to our three major groupings, perhaps as a function of the interview technique employed or the existence of real, but uncommon motivational orientations.

## Discussion

The doctoral learning environment is complex, multi-factorial and continuously evolving. Rather than consisting of a simple system of stable inputs such as supervision, experience or research topic, it is increasingly apparent that doctoral learning involves (p.611) “messy and ambiguous discovery endeavors where students pursue both survival in an unknown territory of new knowledge, and success in the goal-orientated dissertation task” [49]. An increasing suite of interconnected environmental factors including supervision, funding, race, gender, student perceptions, faculty support, socialisation and peer relationships have all been shown to interact with student-related factors to impact upon the learning processes adopted in doctoral studies and the resultant learning outcomes. Complicating our picture of the doctoral learning environment further are data from a number of studies demonstrating that each of these factors likely operates in a context- (discipline, faculty and student) and perception- dependent manner.

We also suggest that these factors are likely to play different roles at different stages of the doctoral candidacy. Amongst these factors, student motivation is accepted as key determinant of learning approaches and eventual success or failure, both in a broad higher education context and in doctoral learning. A unique aspect of motivation is that it not only plays an important role in determining the ways in which a student goes about learning, but it is also highly susceptible to change or influence in response to external stimuli, including the same environmental factors that comprise the doctoral learning environment.

Before concluding as to the implications of the present study to the field of doctoral scholarship, it is important to consider the limitations of the methodology adopted. The present study is based on interview data collected from a relatively small (*n*=17) cohort of self-selected students working in the biomedical sciences at two research intensive universities in Australia. This size and nature of this sample, combined with the semi-structured interview approach is appropriate for the stated aim of this research and consistent with similar approaches to a number of other investigations into doctoral learning [18,19,43,50,51]. Due to restrictions conveyed by a requirement to obtain voluntary participation, any recruitment approach (including that applied in the present study) will convey a response bias, perhaps selecting for those wishing to voice either praise or criticism for their work, missing apathetic students or those simply too engrossed in their work to respond to email solicitations. Interestingly, the cohort in this study represented a broad range of motivational orientations, suggesting that any bias introduced by our choice of recruitment approach was limited.

As discussed, the findings presented herein exhibit parallels with other, previously published data from the United States, Europe and Australia. Despite this, we do stress that generalising the findings of this study to: **i)** other areas of doctoral scholarship (i.e. humanities, social sciences, engineering etc.); **ii)** non-research intensive universities; **iii)** universities outside of Australia (especially in the United States, where doctoral programs are structured quite differently to those in Australia) must naturally be done with a degree of caution. Additionally, it is important to note that although EM and perceptions of external control are associated with sub-optimal learning approaches and outcomes, no attempt was made to correlate the identification of at risk motivational orientations with academic performance or outcome in our study cohort.

We also acknowledge that the data presented herein represent a ‘snap-shot’ of students’ motivational perceptions at a single time-point in their studies. There are good data to suggest that motivational orientation may alter with age [34], and a longitudinal study investigating this phenomenon is likely to yield data of importance to our understanding of doctoral learning processes. Due to the size of this cohort it was not possible to identify potential differences in motivational orientation between students enrolled in different institutions nor at different stages of candidacy. An analysis of motivational orientations, taking these factors into account, would thus constitute an important topic for future research.

In the present study, we identified three clusters of reported motivational orientations (Instrumental, Benevolent Interest and Innate Interest) operating in doctoral students that broadly map to the continuum of IM and EM internalisation proposed in self-determination theory’s organismic integration theory. Stratifying response elements by individual students allowed us to identify three groups of reported motivational orientations in our study cohort.

The identification of an instrumental, self-interested approach as a ubiquitous motivational orientation among doctoral students in the biomedical sciences is, perhaps, not only unsurprising, but also helps support the validity of the data presented in the present study. Self-interest (a predominant factor in the instrumental category) *per se* is widely viewed as a key mediator of human motivation and is a powerful determinant of an individual’s actions [52]. Moreover, much work has been undertaken into the association between university education and career success [53]; in addition, institutional advertising and government policy now places a significant emphasis on vocational training and career advancement as the primary *raison d’etre* for undertaking a course of study at university, likely influencing students’ motivational orientation [7,19].

Our own observations are also in line with previous studies into doctoral learning that suggest an important role for self-interest. Cumming has previously highlighted the importance and interconnectedness of education, training, research, work and career development in the doctoral learning experience [54]; McCuen *et al.* have suggested (p.153) the need for student advisors to “better guide their students in the career building aspects during the Ph.D. process” [55] and more recently, Castro and colleagues have reported data to suggest the importance of career and lifestyle development to motivation in a cohort of female doctoral students in the field of counselling [43].

As discussed above, attrition remains a significant issue with regards the scholarship of doctoral learning. Improving our understanding of the factors that might contribute to doctoral student attrition is of paramount importance in our attempts to improve learning outcomes in this key area of higher education. Of particular interest in the present study is our observation that some 30% of students in this cohort reported an orientation dominated by instrumentalism alone (Students A, B C, H, K) which was characterised by either externally regulated self-interest (money or status) or introjected EM (working to avoid guilt or shame perceived to derive from failure or withdrawal from the program).

Extensive data exists to suggest that as a sole motivational orientation, EM correlates with a surface approach to learning, lowered creativity, reduced task persistence and impaired performance [27-30,32]. These data suggest that the motivational orientations reported by these five students are more likely to be identified in association with sub-optimal learning outcomes and increased risk of attrition. Although the data in the present study are insufficient to draw an association with doctoral attrition in the biomedical sciences, we suggest that the substantial body of data demonstrating an association between EM as a controlling motivational orientation and impaired learning warrant investigation of isolated external motivational orientation in the setting of doctoral attrition.

In contrast, we suggest that the ubiquitous presence of externally motivated self-interest (such as desire to obtain greater financial reward or status) in our cohort of doctoral students is unlikely to be of concern from a learning process and outcome perspective, when it exists in conjunction with, and is balanced by, a well internalised EM or strong IM. Doctoral learning is, as discussed above, a lengthy and complex process. Accordingly, the nature of the challenges that a student has to overcome are likely to vary with the progression of the doctoral program itself, perhaps echoing a key point in Tinto’s landmark paper on student attrition, which highlights the (p.439) “varying difficulties individuals face over time in attempting to persist in college” [56]. The challenges inherent in the initial stages of a doctorate (becoming socialised to the discipline and developing a creative and meaningful research program) are likely to differ greatly from those encountered towards the end (thesis writing, dealing with review criticisms) of the of the process. For example, a student may be well served by a Benevolent Interest motivational orientation alone in the initial stages of his or her work, but without the benefit of an Instrumentalist motivational orientation may also become at risk of attrition should they subsequently come to find (as many doctoral students do) that their work is unlikely to have any direct, translatable benefit to their field of study (e.g.. developing a cure for cancer). Accordingly, a motivational orientation characterised by both task-focused, Instrumentalist (EM) and Benevolent Interest (well internalised EM) may better allow such a student to cope with the evolving nature of challenges across their doctoral studies.

Promisingly, a substantial number (11 of 17) students reported well internalised (benevolent interest) or intrinsic (innate interest) motivational orientations in addition to externally controlled instrumentalism. As discussed previously the importance of a well internalised EM or IM in supporting task persistence and performance is well appreciated [29,32]. In addition to self-interest, the majority of students (F,G,I,L,N,Q) reporting a motivational orientation characterised by benevolent interest also reported possessing an innate interest in their work; a smaller number of students (D,E,J,P) reported only innate interest in association with instrumentalism.

## Conclusions

To date, motivation in the biomedical doctorate has received relatively little attention. In the present study, we aimed to use a semi-structured interview model to advance our understanding of the doctoral learning process by qualitatively describing the motivational orientations reported by doctoral students in the biomedical sciences. Herein, we describe the existence of three clusters of motivational orientation (Instrumentalist, Benevolent Interest, Innate Interest) in biomedical Ph.D. students. These data contribute to our understanding of doctoral learning processes by highlighting differences in student motivational orientation and provide a basis for additional research into this important area of scholarship.

Several conclusions may be drawn from this study. First, and perhaps most promisingly, a significant number of students in our cohort (12 out of 17) reported well internalised or IM motivational orientations that, based on the literature, are likely consistent with deep approaches to learning, task persistence and meaningful learning outcomes. Moreover, 11 of these 17 students also reported adopting an Instrumentalist (EM) motivational orientation in conjunction with well internalised or IM motivational orientations. This is a motivational combination which we suggest may be of benefit in assisting students to cope with the changing nature of doctoral learning processes. The factors (student and environmental) that combine to support (and, importantly, undermine) IM and EM motivational orientations in biomedical Ph.D. students remain poorly defined. However, work from other areas of doctoral scholarship (in particular by Golde, Gardner, Kyndt, the Carnegie Foundation and Cumming) suggests that socialisation to institutional and disciplinary research culture, combined with the promotion of a sense of agency, efficacy and collegiality is key to promoting doctoral success and minimising attrition [1,2,8,11,20]; accordingly, an analysis of these factors against student motivational orientation would seem an important future research question for doctoral learning in the biomedical sciences.

Of particular interest is the potential difference in learning approach, and capacity for persistence that might be associated with the variable presence of benevolent interest and innate interest identified in our cohort. Benevolent interest was characterised by a well internalised EM, wherein the individual has developed a strong personal investment in and affiliation with the task at hand (for example, students reporting that they found a research project important and worthwhile because they had the opportunity to develop treatments for children with cancer). In contrast, innate interest was characterised by intrinsic motivation, that is, the task was enjoyable by and of itself without input from a separable, external contingency (e.g. a cure for cancer). Although outside the scope of the present study, it would be of considerable interest to investigate if this apparent difference in motivational orientation associated with a difference in students’ learning approaches and subsequent differences in learning outcomes. Martin and Marsh, for example, have previously identified five elements that are conducive to academic buoyancy (dealing with the contingencies inherent in every-day academic life), namely (p.365) “control, confidence (high self-efficacy), coordination (high planning), composure (low anxiety) and commitment (high persistence)” [57]. Within the realm of the biomedical doctorate, does having a personal identification with and commitment to the task at hand (i.e. helping sick children) in addition to an innate interest confer benefits such as improved task persistence or better ability to cope with set-backs or failure?

Secondly, a smaller number of students (5 out of 17, approximately 30%) identified motivational orientations characterised by EM alone. It would be of particular interest to attempt to: **i)** identify the underlying reasons (either innate to the student or deriving from that particular students’ learning environment) that may result in students adopting externally regulated motivational orientations towards their doctoral studies; and **ii)** undertake a longitudinal study to determine if the identification of a potential ‘at risk’ motivational orientation in biomedical Ph.D. students correlated with any difference in rates of attrition, time to completion, course satisfaction and post-doctoral career development.

As noted above, the introduction of task-contingent rewards and perceptions of control and competition are associated with the undermining of IM. In line with contemporary developments in the United Kingdom and United States, the contemporary biomedical doctorate in Australia is increasingly characterised by tightly-regulated completion times (control), an emphasis on publishing during one’s time as a student (task-contingent reward) and the increased competition for limited post-doctoral funding [7]. There is also significant debate regarding the lack of tenure-track and faculty jobs available for graduates and even the continued relevance of traditional doctoral programs in an increasingly cross-discipline, output focused research market [58]. In future research in this area it would be of great interest to examine the impact these contemporary environmental factors might play in supporting an EM in biomedical Ph.D. students, and whether or not these factors were impacting doctoral learning outcomes in a positive or negative fashion.

The Cargnegie Foundation’s Initiative on the Doctorate highlighted the importance of regular, honest and clear communication between institutions, staff and students in promoting success in doctoral learning processes [2,8,11,12]. More recently, Brailsford has advocated for reflective discussions between students and staff with regards motivation at the commencement of doctoral studies [19]. In light of the context-sensitive nature of motivation, and the data presented herein, we suggest that regular meetings between students, supervisors and support staff to discuss and reflect on study motivation and approach (and the reasons underlying the adoption of this orientation) may be of benefit to doctoral learning when held across the duration of a student’s doctoral candidacy.

## Competing interests

The authors report no competing interests.

## Authors’ contributions

MWK and EC designed the interview instrument. MWK, TJM and MP arranged and conducted interviews. MWK transcribed and validated interviews. MWK, TJM, MP and EC analysed the data. MWK wrote the manuscript. MWK, TJM, MP and EC reviewed and edited the manuscript. All authors read and approved the final manuscript.

## Pre-publication history

The pre-publication history for this paper can be accessed here:

http://www.biomedcentral.com/1472-6920/14/38/prepub
